# Fatal Tumour Lysis Syndrome Induced by Brigatinib in a Lung Adenocarcinoma Patient Treated With Sequential ALK Inhibitors: A Case Report

**DOI:** 10.3389/fphar.2021.809467

**Published:** 2021-12-20

**Authors:** Yadong Wang, Tiange Wang, Jianchao Xue, Ziqi Jia, Xinyu Liu, Bowen Li, Ji Li, Xiaoguang Li, Weiwei Wang, Zhongxing Bing, Lei Cao, Zhili Cao, Naixin Liang

**Affiliations:** ^1^ Department of Thoracic Surgery, Peking Union Medical College Hospital, Chinese Academy of Medical Sciences and Peking Union Medical College, Beijing, China; ^2^ Chinese Academy of Medical Sciences and Peking Union Medical College, Beijing, China; ^3^ Laser Aesthetic Center, Plastic Surgery Hospital, Chinese Academy of Medical Sciences and Peking Union Medical College, Beijing, China; ^4^ Eight-Year MD Program, Chinese Academy of Medical Sciences and Peking Union Medical College, Beijing, China; ^5^ Department of Pathology, Peking Union Medical College Hospital, Chinese Academy of Medical Sciences and Peking Union Medical College, Beijing, China; ^6^ Minimally Invasive Tumor Therapies Center, Beijing Hospital, National Center of Gerontology, Institute of Geriatric Medicine, Chinese Academy of Medical Sciences, Beijing, China

**Keywords:** tumour lysis syndrome, non-small cell lung cancer, targeted therapy, brigatinib, acute kidney injury, case report

## Abstract

Tumour lysis syndrome (TLS) represents a group of fatal metabolic derangements resulting from the rapid breakdown of tumour cells. TLS typically occurs soon after the administration of chemotherapy in haematologic malignancies but is rarely observed in solid tumours. Here, we report a case of brigatinib-induced TLS after treatment with sequential anaplastic lymphoma kinase (*ALK*) inhibitors in a patient with advanced *ALK*-rearranged lung adenocarcinoma. The patient was treated sequentially with crizotinib, alectinib, and ensartinib. High-throughput molecular profiling after disease progression indicated that brigatinib may overcome *ALK* resistance mutations, so the patient was administered brigatinib as the fourth-line treatment. After 22 days of therapy, he developed oliguria, fever, and progressive dyspnoea. Clinical manifestations and laboratory findings met the diagnostic criteria for TLS. The significant decrease in the abundance of *ALK* mutations in plasma indicated a therapeutic response at the molecular level. Consequently, the diagnosis of brigatinib-induced TLS was established. To the best of our knowledge, this is the first case of TLS induced by sequential targeted therapy in non-small cell lung cancer. With the extensive application of sequential therapy with more potent next-generation targeted therapeutic drugs, special attention should be given to this rare but severe complication.

## Introduction

Tumour lysis syndrome (TLS) is a life-threatening oncology emergency. It is induced by a massive release of intracellular contents into the circulation when tumour cells breakdown spontaneously or after the initiation of chemotherapy (Howard et al., 2011). TLS mostly develops in the context of haematologic malignancies and is relatively rare in solid tumours (Cairo et al., 2010). Only a few cases of TLS have been reported in non-small cell lung cancer (NSCLC), most of which are induced by chemotherapy (Myint et al., 2015).

With the development of molecular diagnoses and targeted therapies, small molecular tyrosine kinase inhibitors (TKIs) have become the cornerstone of treatment for patients with targetable mutations. The most common therapeutic targets include epidermal growth factor receptor (*EGFR*) and anaplastic lymphoma kinase (*ALK*) genomic alterations in NSCLC (Recondo et al., 2018). Over the past years, *EGFR*-TKIs and *ALK*-TKIs have continuously evolved from the first generation to the third generation (Mok et al., 2009; Solomon et al., 2014; Ramalingam et al., 2020; Shaw et al., 2020). In patients with sensitizing mutations who experienced progression after first-line targeted therapy, sequential treatment with more potent next-generation inhibitors is recommended to overcome drug resistance and improve patient outcomes (Mok et al., 2017; Shaw et al., 2019; Nishio et al., 2021). However, the usage of more potent next-generation inhibitors may increase the risk of rapid breakdown of tumour cells and the incidence of TLS. In this study, we describe a patient with metastatic *ALK*-rearranged NSCLC who received multiple prior *ALK* inhibitors during his treatment course and subsequently died from brigatinib-induced TLS.

## Case Presentation

**FIGURE 1:**
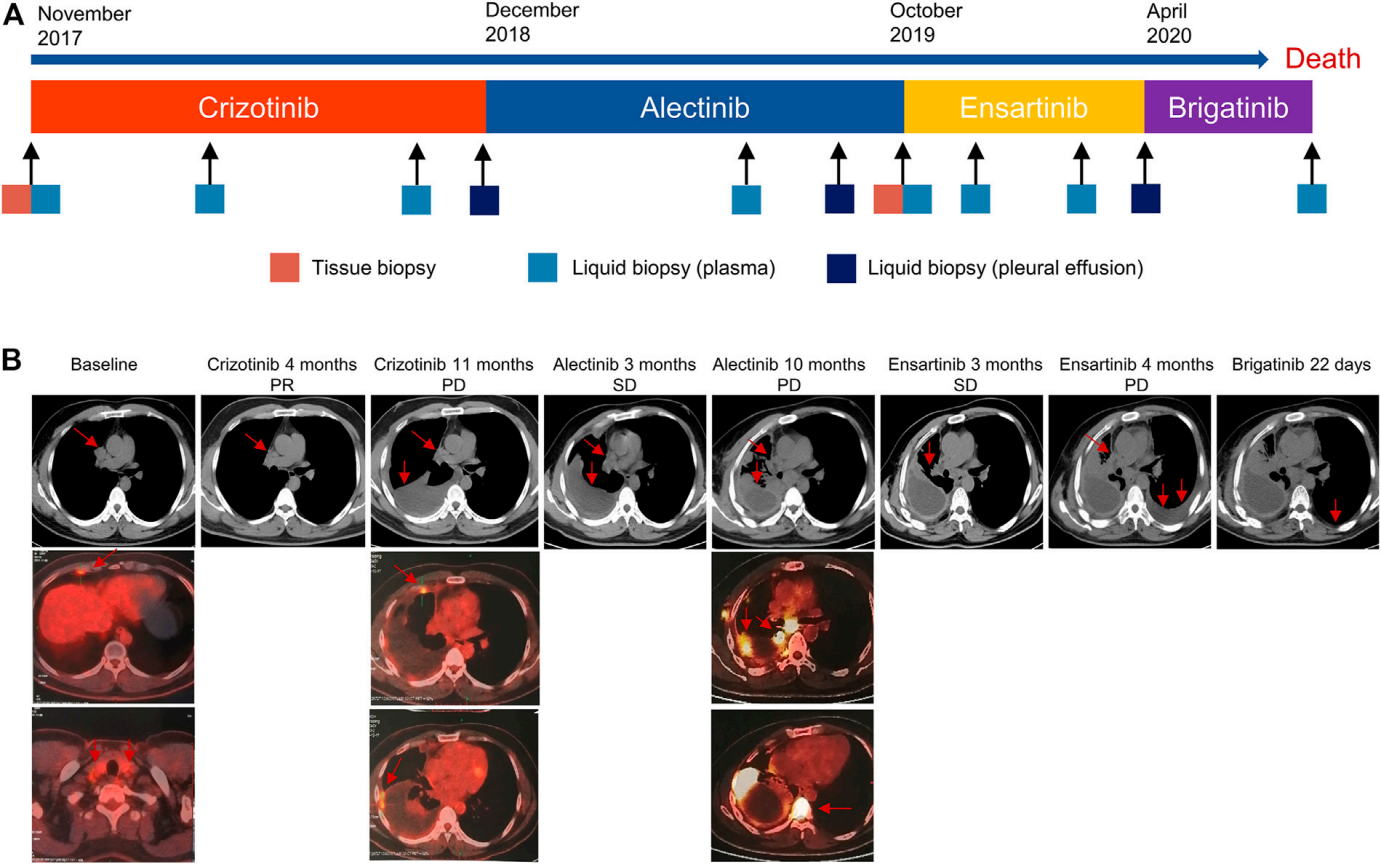
The Clinical course of the disease, treatment history, and response evaluation. **(A)** Timeline of treatment and molecular profiling based on tissue and liquid biopsies. **(B)** Duration of disease response evaluated by CT and PET/CT. CT, computed tomography; PET/CT, positron-emission tomography/computed tomography.

**FIGURE 2:**
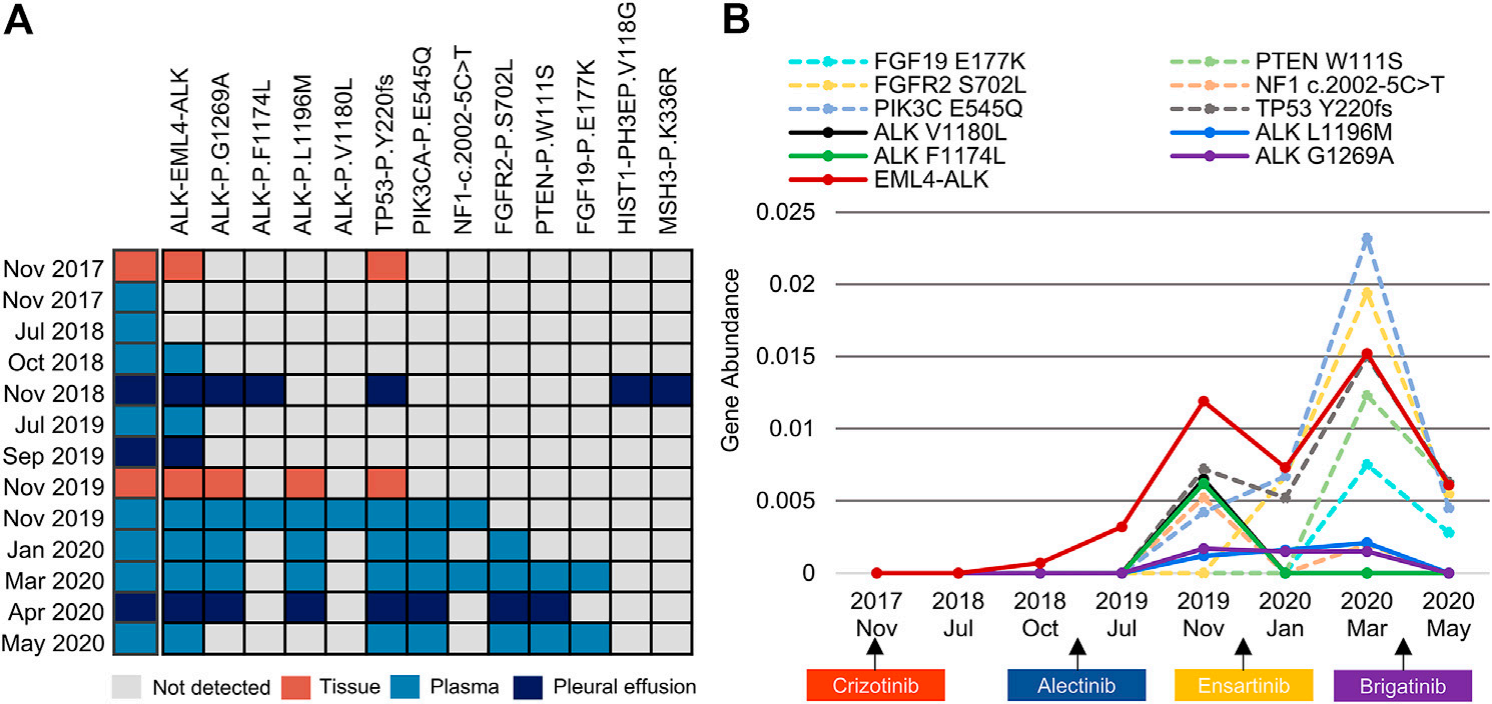
An overview of somatic mutation profiles within tissue and liquid biopsies using the next-generation sequencing technique. **(A)** Each row represents one individual biopsy sample, and each column represents one somatic genetic alteration. **(B)** Dynamic changes in the gene abundance of plasma ctDNA. Solid lines represent ALK mutations, and dashed lines represent concomitant mutations. ctDNA, circulating tumour DNA.

A 39-year-old nonsmoking male presented to our hospital with chest pain in November 2017 (Figure 1A). The patient had no significant medical or family history of malignancy. Chest computed tomography (CT) demonstrated a 26-mm solid nodule in the hilum of the right lung (Figure 1B). Positron-emission tomography/computed tomography (PET/CT) revealed multiple right pleural and bilateral supraclavicular lymph node metastases without distant metastasis. The patient was diagnosed with lung adenocarcinoma (pT4N3M1a, stage IVA) following a tissue biopsy via video-assisted thoracoscopic surgery (VATS). Next-generation sequencing (NGS) with a 168-gene sequencing panel (Burning Rock, Guangzhou, China) revealed echinoderm microtubule-associated protein-like 4 (*EML4*)*-ALK* rearrangement and *TP53* mutation (Figure 2A). The patient was administered crizotinib (250 mg twice daily) as the first-line treatment and achieved a partial response according to the Response Evaluation Criteria in Solid Tumors (RECIST 1.1). After effective treatment for 11 months, he was admitted to our hospital again for dyspnoea. CT showed a large right pleural effusion. Thoracentesis was performed to collect pleural effusion for circulating tumour DNA (ctDNA) analysis. NGS identified two emerging mutations in the *ALK* kinase domain, F1174L and G1269A, indicating the onset of resistance to crizotinib.

Crizotinib was discontinued, and he was administered alectinib (600 mg twice daily) as the second-line treatment. Stable disease (SD) was maintained for 10 months, during which only *EML4-ALK* was detectable in the plasma and pleural effusion. The patient gradually developed low back pain. PET/CT demonstrated bone metastasis and systemic progression. VATS biopsy was performed again, and NGS of metastatic tissues identified *ALK* L1196M and G1269A mutations in addition to *EML4-ALK* rearrangement and *TP53* mutation. Meanwhile, plasma ctDNA revealed the other two *ALK* F1174L and V1180L mutations. Considering disease progression and drug accessibility, he was switched to ensartinib (225 mg once daily) as the third-line treatment. Subsequent CT confirmed encapsulated pleural effusion after 3 months of therapy, and he had SD that lasted for 4 months. Although the *ALK* F1174L and V1180L mutations were subsequently not detectable in plasma, the mutational spectrum became significantly complicated with the emergence of various concomitant mutations.

In April 2020, the patient experienced progressive left pleural effusion and started receiving brigatinib (90 mg once daily and then 180 mg once daily at 1 week) as the fourth-line treatment. Symptoms or laboratory abnormalities associated with TLS were not observed before initiation of brigatinib except for slightly elevated lactate dehydrogenase (Table 1). On Day 6, he reported mild relief from dyspnoea, and brigatinib was continued. However, the patient presented to the emergency department with oliguria, fever, and progressive dyspnoea after 22 days of therapy. The electrocardiogram revealed atrial fibrillation. Laboratory tests revealed the following results: hyperuricaemia (956 μmol/L), hyperkalaemia (6.27 mmol/L), hyperphosphatemia (2.65 mmol/L), hypocalcaemia (1.93 mmol/L), elevated urea nitrogen (36.4 mmol/L), creatinine (320.2 μmol/L), and lactate dehydrogenase (1,477 U/L). The estimated glomerular filtration rate (eGFR) was 19.5 ml/min/1.73 m^2^. Dynamic monitoring of plasma ctDNA revealed a significant decrease in the abundance of *EML4-ALK* rearrangement from 1.52 to 0.61%. The other resistance-associated *ALK* point mutations disappeared (Figure 2B).

**TABLE 1:** Laboratory parameters before (Day -12) and after (Day 22) brigatinib treatment.

Parameters	Reference range	Before (day -12)	After (day 22)
Potassium (mmol/L)	3.5–5.5	4.9	6.27
Phosphorous (mmol/L)	0.81–1.45	1.39	2.65
Calcium (mmol/L)	2.13–2.70	2.32	1.93
Uric acid (μmol/L)	210–416	363	956
LDH (U/L)	0–250	376	1477
BUN (mmol/L)	2.78–7.14	5.29	36.4
Creatinine (μmol/L)	59–104	73	320.2
eGFR (ml/min/1.73 m^2^)	80–120	108.6	19.5

LDH, lactate dehydrogenase; BUN, blood urea nitrogen; eGFR, estimated glomerular filtration rate.

Based on clinical manifestations and laboratory findings, the diagnosis of brigatinib-induced TLS was established. The patient immediately received intubation and mechanical ventilation for progressive respiratory failure. He was subsequently treated with vigorous volume expansion, torasemide, sodium polystyrene sulfonate, calcium gluconate, and ceftazidime. Despite these aggressive medical interventions, his clinical and biochemical parameters did not improve, and continuous renal replacement therapy was required. The patient and his family refused emergent haemodialysis or further interventions due to the dismal prognosis. He was transitioned to comfort care and passed away within 24 h.

## Discussion

TLS is a potentially lethal complication in the therapy of tumours with a mortality rate of up to 35% in solid tumours (Baeksgaard and Sørensen, 2003). TLS occurs frequently in patients with haematologic malignancies after the initiation of cytotoxic chemotherapy, such as acute lymphoblastic leukaemia and high-grade non-Hodgkin’s lymphoma (Cairo and Bishop, 2004). An increasing number of TLS cases have been reported in patients with solid tumours that were previously rarely associated with this complication (Mirrakhimov et al., 2014). Moreover, with the advent of more potent targeted agents, TLS induced by targeted therapy has been reported in several solid tumours, such as melanoma and colorectal, liver, kidney, and breast cancers (Krishnan et al., 2008; Huang and Yang, 2009; Michels et al., 2010; Handy et al., 2021; Tachibana et al., 2021). To the best of our knowledge, this is the first case of TLS induced by targeted therapy in NSCLC.

The pathophysiological mechanisms of TLS are mainly due to the rapid lysis of tumour cells with a massive release of intracellular contents, such as potassium, phosphorus, and nucleic acids that are ultimately metabolized to uric acids (Howard et al., 2011). These chemical substances are mainly excreted through the kidney, which exceeds the compensatory capacity of the kidney and leads to metabolic disorders and electrolyte disturbances. The most commonly employed criteria of TLS diagnosis were developed by Cairo and Bishop in 2004 (Cairo and Bishop, 2004). In their classification system, TLS is classified as laboratory TLS or clinical TLS. Laboratory TLS requires at least two of the following laboratory abnormalities within 3 days before or up to 7 days after the initiation of therapy: hyperuricaemia, hyperkalaemia, hyperphosphatemia, and hypocalcaemia. Clinical TLS is defined as the presence of laboratory TLS and at least one of the following clinical complications: renal insufficiency, seizures, and cardiac arrhythmias or sudden death (Cairo and Bishop, 2004). In our case, uric acid, potassium, and phosphate levels met the criteria for laboratory TLS, and the creatine level, atrial fibrillation, and death met the criteria for clinical TLS.

Brigatinib is a potent next-generation *ALK* inhibitor with demonstrated activity against *ALK* rearrangement and multiple mutations in the *ALK* kinase domain that confer resistance (Kim et al., 2017; Camidge et al., 2018; Camidge et al., 2020; Nishio et al., 2021; Stinchcombe et al., 2021). In our case, the patient was treated with crizotinib as the first-line treatment and subsequently switched to alectinib due to disease progression. Stable disease was maintained for 10 months, after which the patient gradually developed bone metastasis and systemic progression. NGS identified acquired *ALK* resistance mutations, including F1174L, G1269A, L1196M, and V1180L. At that time, lorlatinib was not approved in China, and the patient declined platinum-doublet chemotherapy. Yang et al. reported that ensartinib could exhibit clinical activity in patients with acquired resistance mutations to some other second-generation *ALK* TKIs (Yang et al., 2020; Yang et al., 2021). Therefore, the patient was administered ensartinib as the third-line treatment and subsequently experienced progressive left pleural effusion. The phase 2 J-ALTA trial confirmed the antitumour activity of brigatinib in patients with *ALK*-rearranged NSCLC refractory to alectinib or other *ALK*-TKIs (Nishio et al., 2021). Previous literature reported that brigatinib may overcome acquired *ALK* resistance mutations in this patient, so the patient chose brigatinib as the fourth-line treatment (Gainor et al., 2016; Zhang et al., 2016; Recondo et al., 2018; Gristina et al., 2020).

During the patient’s treatment course, repeat molecular profiling based on tissue or liquid biopsies played an important role in monitoring the evolution of resistance and tailoring individualized treatment after disease progression (Horn et al., 2019). In recent years, liquid biopsy has gradually become an alternative or complementary approach to tissue biopsy for diagnostic, predictive, and prognostic purposes. Compared with traditional tissue biopsy, liquid biopsy has notable advantages, such as its minimally invasive nature, ability to reflect tumour heterogeneity, and capacity to be repeatedly performed to realize dynamic monitoring of the disease (Russo et al., 2021). Liquid biopsy is particularly suitable for patients for whom sufficient tumour tissues cannot be obtained for molecular profiling. However, liquid biopsy exhibits various limitations due to both biological and technological issues. Foremost among them is the risk of a false-negative result, which might affect subsequent clinical decisions and outcomes. In addition, some other limitations, such as discordance with tissue specimens, remain to be further addressed by technical advances and the establishment of standard processes. In this patient, dynamic monitoring of plasma ctDNA revealed a significant decrease in the abundance of *EML4-ALK* rearrangement and the disappearance of other resistance-associated *ALK* point mutations. It is difficult to directly evaluate the effect of brigatinib because the patient only received it for less than 1 month. However, the significant decrease or clearance of *ALK* mutations after treatment with brigatinib indicated a therapeutic response at the molecular level (Wang et al., 2018). Therefore, the diagnosis of brigatinib-induced TLS was established after carefully excluding other possible contributing factors.

The existing Cairo-Bishop criteria were mainly based on the clinical features and treatment paradigms of haematologic malignancies. Thus, it is less suited for solid tumours and new treatment modalities, such as targeted therapy and immunotherapy. The Cairo-Bishop criteria have several limitations and need to be further improved. First, receipt of chemotherapy is the basic foundation for the diagnosis of TLS. However, TLS can develop spontaneously or after the initiation of surgery, radiotherapy, targeted therapy, and immunotherapy in solid tumours (Noh et al., 2008; Shenoy, 2009; Shin et al., 2019; Sugimoto et al., 2020; Tachibana et al., 2021). With the emergence of more potent therapeutic modalities and multiple combined therapies, especially in advanced tumours, predisposing factors of TLS should not just be limited to chemotherapy. Second, the time of occurrence of TLS in the Cairo-Bishop criteria may need to be redefined. Most conventional chemotherapy agents kill tumour cells through cytotoxic effects with rapid onset of action. Therefore, chemotherapy-induced TLS was diagnosed relatively early. Nevertheless, the mechanism of chemotherapy is considerably different from that of targeted therapy and immunotherapy. Targeted therapy acts specifically on certain signalling pathways and precisely kills tumour cells (Recondo et al., 2018). The principle of immunotherapy is to activate the patient’s own immune system to attack malignant tumour cells (Alsaab et al., 2017). Consequently, these therapeutic agents may have a slower onset of efficacy and relatively moderate response at the beginning of therapy compared with chemotherapy. Nicholaou et al. reported a case of sunitinib-induced TLS in a patient with kidney renal clear cell carcinoma after 14 days of treatment (Nicholaou et al., 2007). Huang et al. reported a case of sorafenib-induced TLS after 30 days of treatment in an advanced hepatocellular carcinoma patient (Huang and Yang, 2009). In our case, the patient developed TLS after 22 days of brigatinib treatment. These results indicate that the Cairo-Bishop criteria may potentially exclude some patients who develop TLS over a 7-days period after initiating treatment. Therefore, the Cairo-Bishop criteria need to be dynamically updated to adapt the features of novel treatment strategies.

Given the seriousness and urgency of the TLS, early prevention and early recognition are vital for reducing mortality. Major risk factors for TLS include but are not limited to a large tumour burden, tumours with a high proliferative rate, highly effective antitumour treatment, liver metastases, and baseline renal insufficiency (Amiri, 2015). According to these risk factors, patients can be divided into low-risk, intermediate-risk, and high-risk groups, which is of central importance to enable stratified management. Prevention measures include adequate hydration and prophylactic administration of rasburicase in high-risk patients, adequate hydration plus allopurinol or rasburicase for intermediate-risk patients, and careful monitoring for low-risk patients (Coiffier et al., 2008; Cairo et al., 2010). Additionally, repeat dynamic detection of laboratory and clinical indicators before and during treatment is recommended. In our case, the patient was at an advanced stage with multiple metastases and had been sequentially treated with crizotinib, alectinib, and ensartinib before initiation of brigatinib. Therefore, multiline TKI therapy and a high tumour load may be associated with rapid lysis of tumour cells and the occurrence of TLS when the patient was treated with a more potent next-generation TKI. Actually, with the extensive application of sequential therapy with potent next-generation TKIs, such as osimertinib and lorlatinib, this risk factor should attract sufficient attention from clinicians (Ramalingam et al., 2020; Shaw et al., 2020). In addition, patients with NSCLC typically receive oral small molecular TKIs at home instead of at the hospital. Thus, these patients require careful observation and appropriate laboratory tests in the initial period of targeted therapy.

As soon as symptoms or laboratory abnormalities associated with TLS are observed, multidisciplinary diagnostic assessment and strict monitoring are necessary. Accurate and timely diagnosis of TLS is a prerequisite for taking effective measures to prevent the disease from further worsening and reduce overall mortality. Once a definite diagnosis of TLS has been established, antineoplastic treatment that may lead to TLS should be discontinued. Frequent monitoring examinations involving continuous electrocardiogram, repeat blood biochemistry parameters and urine analysis should be performed. The patient should be monitored and treated in the intensive care unit with an experienced multidisciplinary team according to the standard guidelines for TLS (Jones et al., 2015).

Another noteworthy concern is whether molecular targeted drugs could be restarted after recovery from TLS. In clinical practice, the decision must be made regarding the potential clinical benefit versus the relative risk of the re-emergence of TLS. Several studies reported that readministration of molecular targeted drugs at a reduced dose could confer a survival benefit with no TLS recurrence (Joshita et al., 2010; Michels et al., 2010; Shimizu et al., 2021). Therefore, resumption of prior treatment may be attempted with careful monitoring if the targeted drug is irreplaceable or significantly more effective than the alternatives.

## Conclusion

In summary, our case indicated that TLS can be induced at a relatively late timing by sequential targeted therapy in NSCLC. Given the seriousness of TLS and the extensive use of sequential targeted therapy, clinicians should maintain a high clinical suspicion for TLS and take appropriate measures to reduce mortality.

## Data Availability

The original contributions presented in the study are included in the article/supplementary material, further inquiries can be directed to the corresponding author.

## References

[B1] Alsaab H. O. Sau S. Alzhrani R. Tatiparti K. Bhise K. Kashaw S. K. (2017). PD-1 and PD-L1 Checkpoint Signaling Inhibition for Cancer Immunotherapy: Mechanism, Combinations, and Clinical Outcome. Front. Pharmacol. 8, 561. 10.3389/fphar.2017.00561 28878676 PMC5572324

[B2] Amiri F. S. (2015). Concurrent Acute Spontaneous Tumor Lysis Syndrome Complicated with Multiple Organ Failure in a Patient with Pre-existing Undiagnosed Lung Cancer. CEN Case Rep. 4 (2), 233–237. 10.1007/s13730-015-0175-0 28509107 PMC5413772

[B3] Baeksgaard L. Sørensen J. B. (2003). Acute Tumor Lysis Syndrome in Solid Tumors-Aa Case Report and Review of the Literature. Cancer Chemother. Pharmacol. 51 (3), 187–192. 10.1007/s00280-002-0556-x 12655435

[B4] Cairo M. S. Bishop M. (2004). Tumour Lysis Syndrome: New Therapeutic Strategies and Classification. Br. J. Haematol. 127 (1), 3–11. 10.1111/j.1365-2141.2004.05094.x 15384972

[B5] Cairo M. S. Coiffier B. Reiter A. Younes A. (2010). Recommendations for the Evaluation of Risk and Prophylaxis of Tumour Lysis Syndrome (TLS) in Adults and Children with Malignant Diseases: An Expert TLS Panel Consensus. Br. J. Haematol. 149 (4), 578–586. 10.1111/j.1365-2141.2010.08143.x 20331465

[B6] Camidge D. R. Kim H. R. Ahn M. J. Yang J. C. Han J. Y. Lee J. S. (2018). Brigatinib Versus Crizotinib in ALK-Positive Non-Small-Cell Lung Cancer. N. Engl. J. Med. 379 (21), 2027–2039. 10.1056/NEJMoa1810171 30280657

[B7] Camidge D. R. Kim H. R. Ahn M. J. Yang J. C. H. Han J. Y. Hochmair M. J. (2020). Brigatinib Versus Crizotinib in Advanced ALK Inhibitor-Naive ALK-Positive Non-Small Cell Lung Cancer: Second Interim Analysis of the Phase III ALTA-1L Trial. J. Clin. Oncol. 38 (31), 3592–3603. 10.1200/jco.20.00505 32780660 PMC7605398

[B8] Coiffier B. Altman A. Pui C. H. Younes A. Cairo M. S. (2008). Guidelines for the Management of Pediatric and Adult Tumor Lysis Syndrome: An Evidence-Based Review. J. Clin. Oncol. 26 (16), 2767–2778. 10.1200/jco.2007.15.0177 18509186

[B9] Gainor J. F. Dardaei L. Yoda S. Friboulet L. Leshchiner I. Katayama R. (2016). Molecular Mechanisms of Resistance to First- and Second-Generation ALK Inhibitors in ALK-Rearranged Lung Cancer. Cancer Discov. 6 (10), 1118–1133. 10.1158/2159-8290.Cd-16-0596 27432227 PMC5050111

[B10] Gristina V. La Mantia M. Iacono F. Galvano A. Russo A. Bazan V. (2020). The Emerging Therapeutic Landscape of ALK Inhibitors in Non-Small Cell Lung Cancer. Pharmaceuticals (Basel) 13 (12), 474. 10.3390/ph13120474 PMC7766858 33352844

[B11] Handy C. Wesolowski R. Gillespie M. Lause M. Sardesai S. Williams N. (2021). Tumor Lysis Syndrome in a Patient with Metastatic Breast Cancer Treated with Alpelisib. Breast Cancer (Auckl) 15, 117822342110374. 10.1177/11782234211037421 PMC8408891 34483661

[B12] Horn L. Whisenant J. G. Wakelee H. Reckamp K. L. Qiao H. Leal T. A. (2019). Monitoring Therapeutic Response and Resistance: Analysis of Circulating Tumor DNA in Patients With ALK+ Lung Cancer. J. Thorac. Oncol. 14 (11), 1901–1911. 10.1016/j.jtho.2019.08.003 31446141 PMC6823161

[B13] Howard S. C. Jones D. P. Pui C. H. (2011). The Tumor Lysis Syndrome. N. Engl. J. Med. 364 (19), 1844–1854. 10.1056/NEJMra0904569 21561350 PMC3437249

[B14] Huang W. S. Yang C. H. (2009). Sorafenib Induced Tumor Lysis Syndrome in an Advanced Hepatocellular Carcinoma Patient. World J. Gastroenterol. 15 (35), 4464–4466. 10.3748/wjg.15.4464 19764104 PMC2747073

[B15] Jones G. L. Will A. Jackson G. H. Webb N. J. Rule S. (2015). Guidelines for the Management of Tumour Lysis Syndrome in Adults and Children with Haematological Malignancies on Behalf of the British Committee for Standards in Haematology. Br. J. Haematol. 169 (5), 661–671. 10.1111/bjh.13403 25876990

[B16] Joshita S. Yoshizawa K. Sano K. Kobayashi S. Sekiguchi T. Morita S. (2010). A Patient with Advanced Hepatocellular Carcinoma Treated with Sorafenib Tosylate Showed Massive Tumor Lysis with Avoidance of Tumor Lysis Syndrome. Intern. Med. 49 (11), 991–994. 10.2169/internalmedicine.49.3153 20519814

[B17] Kim D. W. Tiseo M. Ahn M. J. Reckamp K. L. Hansen K. H. Kim S. W. (2017). Brigatinib in Patients With Crizotinib-Refractory Anaplastic Lymphoma Kinase-Positive Non-Small-Cell Lung Cancer: A Randomized, Multicenter Phase II Trial. J. Clin. Oncol. 35 (22), 2490–2498. 10.1200/jco.2016.71.5904 28475456

[B18] Krishnan G. D'Silva K. Al-Janadi A. (2008). Cetuximab-Related Tumor Lysis Syndrome in Metastatic colon Carcinoma. J. Clin. Oncol. 26 (14), 2406–2408. 10.1200/jco.2007.14.7603 18467734

[B19] Michels J. Lassau N. Gross-Goupil M. Massard C. Mejean A. Escudier B. (2010). Sunitinib Inducing Tumor Lysis Syndrome in a Patient Treated for Renal Carcinoma. Invest. New Drugs 28 (5), 690–693. 10.1007/s10637-009-9275-z 19547920

[B20] Mirrakhimov A. E. Ali A. M. Khan M. Barbaryan A. (2014). Tumor Lysis Syndrome in Solid Tumors: An up to Date Review of the Literature. Rare Tumors 6 (2), 5389. 10.4081/rt.2014.5389 25002953 PMC4083673

[B21] Mok T. S. Wu Y-L. Ahn M-J. Garassino M. C. Kim H. R. Ramalingam S. S. (2017). Osimertinib or Platinum-Pemetrexed in EGFR T790M-Positive Lung Cancer. N. Engl. J. Med. 376 (7), 629–640. 10.1056/NEJMoa1612674 27959700 PMC6762027

[B22] Mok T. S. Wu Y. L. Thongprasert S. Yang C. H. Chu D. T. Saijo N. (2009). Gefitinib or Carboplatin-Paclitaxel in Pulmonary Adenocarcinoma. N. Engl. J. Med. 361 (10), 947–957. 10.1056/NEJMoa0810699 19692680

[B23] Myint Z. W. Verla-Tebit E. Cho B. B. Goodner S. A. Stelow E. B. Weiss G. R. (2015). Tumor Lysis Syndrome in a Patient with Metastatic Non-small Cell Lung Cancer: Case Report and Literature Review. Cancer Treat. Commun. 4, 10–14. 10.1016/j.ctrc.2015.03.002

[B24] Nicholaou T. Wong R. Davis I. D. (2007). Tumour Lysis Syndrome in a Patient with Renal-Cell Carcinoma Treated with Sunitinib Malate. Lancet 369 (9577), 1923–1924. 10.1016/s0140-6736(07)60903-9 17560435

[B25] Nishio M. Yoshida T. Kumagai T. Hida T. Toyozawa R. Shimokawaji T. (2021). Brigatinib in Japanese Patients With ALK-Positive NSCLC Previously Treated With Alectinib and Other Tyrosine Kinase Inhibitors: Outcomes of the Phase 2 J-ALTA Trial. J. Thorac. Oncol. 16 (3), 452–463. 10.1016/j.jtho.2020.11.004 33248320

[B26] Noh G. Y. Choe D. H. Kim C. H. Lee J. C. (2008). Fatal Tumor Lysis Syndrome during Radiotherapy for Non-small-cell Lung Cancer. J. Clin. Oncol. 26 (36), 6005–6006. 10.1200/jco.2008.19.4308 19029410

[B27] Ramalingam S. S. Vansteenkiste J. Planchard D. Cho B. C. Gray J. E. Ohe Y. (2020). Overall Survival with Osimertinib in Untreated, EGFR-Mutated Advanced NSCLC. N. Engl. J. Med. 382 (1), 41–50. 10.1056/NEJMoa1913662 31751012

[B28] Recondo G. Facchinetti F. Olaussen K. A. Besse B. Friboulet L. (2018). Making the First Move in EGFR-Driven or ALK-Driven NSCLC: First-Generation or Next-Generation TKI? Nat. Rev. Clin. Oncol. 15 (11), 694–708. 10.1038/s41571-018-0081-4 30108370

[B29] Russo A. Incorvaia L. Del Re M. Malapelle U. Capoluongo E. Gristina V. (2021). The Molecular Profiling of Solid Tumors by Liquid Biopsy: A Position Paper of the AIOM-SIAPEC-IAP-SIBioC-SIC-SIF Italian Scientific Societies. ESMO Open 6 (3), 100164. 10.1016/j.esmoop.2021.100164 34091263 PMC8182269

[B30] Shaw A. T. Bauer T. M. de Marinis F. Felip E. Goto Y. Liu G. (2020). First-Line Lorlatinib or Crizotinib in Advanced ALK-Positive Lung Cancer. N. Engl. J. Med. 383 (21), 2018–2029. 10.1056/NEJMoa2027187 33207094

[B31] Shaw A. T. Solomon B. J. Besse B. Bauer T. M. Lin C. C. Soo R. A. (2019). ALK Resistance Mutations and Efficacy of Lorlatinib in Advanced Anaplastic Lymphoma Kinase-Positive Non-small-Cell Lung Cancer. J. Clin. Oncol. 37 (16), 1370–1379. 10.1200/jco.18.02236 30892989 PMC6544460

[B32] Shenoy C. (2009). Acute Spontaneous Tumor Lysis Syndrome in a Patient with Squamous Cell Carcinoma of the Lung. QJM 102 (1), 71–73. 10.1093/qjmed/hcn129 18829711

[B33] Shimizu Y. Sunagozaka H. Yamagata K. Hirai H. Miura M. Yonemoto Y. (2021). Lenvatinib-Induced Tumor Lysis Syndrome in a Patient with Advanced Hepatocellular Carcinoma: A Case Report. Clin. J. Gastroenterol. 14 (2), 645–649. 10.1007/s12328-020-01306-1 33389590

[B34] Shin T. H. Inagaki E. Ganta T. Hartshorn K. Litle V. R. Suzuki K. (2019). Tumor Lysis Syndrome After Bilobectomy for Typical Carcinoid Tumor of the Lung. Ann. Thorac. Surg. 107 (3), e199–e201. 10.1016/j.athoracsur.2018.06.089 30218665

[B35] Solomon B. J. Mok T. Kim D. W. Wu Y. L. Nakagawa K. Mekhail T. (2014). First-Line Crizotinib Versus Chemotherapy in ALK-Positive Lung Cancer. N. Engl. J. Med. 371 (23), 2167–2177. 10.1056/NEJMoa1408440 25470694

[B36] Stinchcombe T. E. Doebele R. C. Wang X. Gerber D. E. Horn L. Camidge D. R. (2021). Preliminary Clinical and Molecular Analysis Results From a Single-Arm Phase 2 Trial of Brigatinib in Patients with Disease Progression After Next-Generation ALK Tyrosine Kinase Inhibitors in Advanced ALK+ NSCLC. J. Thorac. Oncol. 16 (1), 156–161. 10.1016/j.jtho.2020.09.018 33039599

[B37] Sugimoto S. Terashima T. Yamashita T. Iida N. Kitahara M. Hodo Y. (2020). Tumor Lysis Syndrome in a Patient with Metastatic Melanoma Treated with Nivolumab. Clin. J. Gastroenterol. 13 (5), 935–939. 10.1007/s12328-020-01164-x 32594423

[B38] Tachibana K. Ohe S. Tanaka M. Maniwa T. Isei T. (2021). Tumor Lysis Syndrome Induced by BRAF/MEK Double Blockade in a Patient with Metastatic Melanoma: A First Case Report. J. Dermatol. 48 (7), e324–e326. 10.1111/1346-8138.15894 33990995

[B39] Wang Z. Cheng Y. An T. Gao H. Wang K. Zhou Q. (2018). Detection of EGFR Mutations in Plasma Circulating Tumour DNA as a Selection Criterion for First-Line Gefitinib Treatment in Patients with Advanced Lung Adenocarcinoma (BENEFIT): a Phase 2, Single-Arm, Multicentre Clinical Trial. Lancet Respir. Med. 6 (9), 681–690. 10.1016/s2213-2600(18)30264-9 30017884

[B40] Yang Y. Huang J. Wang T. Zhou J. Zheng J. Feng J. (2021). Decoding the Evolutionary Response to Ensartinib in Patients With ALK-Positive NSCLC by Dynamic Circulating Tumor DNA Sequencing. J. Thorac. Oncol. 16 (5), 827–839. 10.1016/j.jtho.2021.01.1615 33588113

[B41] Yang Y. Zhou J. Zhou J. Feng J. Zhuang W. Chen J. (2020). Efficacy, Safety, and Biomarker Analysis of Ensartinib in Crizotinib-Resistant, ALK-Positive Non-Small-Cell Lung Cancer: A Multicentre, Phase 2 Trial. Lancet Respir. Med. 8 (1), 45–53. 10.1016/s2213-2600(19)30252-8 31628085

[B42] Zhang S. Anjum R. Squillace R. Nadworny S. Zhou T. Keats J. (2016). The Potent ALK Inhibitor Brigatinib (AP26113) Overcomes Mechanisms of Resistance to First- and Second-Generation ALK Inhibitors in Preclinical Models. Clin. Cancer Res. 22 (22), 5527–5538. 10.1158/1078-0432.Ccr-16-0569 27780853

